# Medical Imaging Technologists in Radiomics Era: An Alice in Wonderland Problem

**Published:** 2019-01

**Authors:** Hamid ABDOLLAHI, Isaac SHIRI, Mohammad HEYDARI

**Affiliations:** 1. Dept. of Medical Physics, School of Medicine, Iran University of Medical Sciences, Tehran, Iran; 2. Research Center for Molecular and Cellular Imaging, Tehran University of Medical Sciences, Tehran, Iran; 3. Biomedical and Health Informatics, Rajaie Cardiovascular Medical and Research Center, Iran University of Medical Sciences, Tehran, Iran; 4. Student Research Committee, School of Health Management and Information Sciences Branch, Iran University of Medical Sciences, Tehran, Iran

## Dear Editor-in-Chief

Radiomics is a new branch of imaging science which aims to extract mineable data from medical images and correlate them to clinical data. It is an advanced approach and has several main stages and substages full of challenges and uncertainties. Radiomics is coming to maturity. After a teething period, a considerable progress is currently being made. In radiomics, a wide range of specialists are involved for data acquisition, presentation, and analysis. Radiology, Oncology, Medical Physics, Medical Engineering, Bioinformatics, Data Science, Biostatistics and many different sciences are involved in final radiomics outcomes. These specialists change the radiomics results directly or indirectly, intentional or unintentional.

There have been several investigations regard to the applications of radiomics from bench to bedside (1, 2). The false discovery rates in radiomics results originated from data uncertainties have resulted in difficulties in clinical decision making (3, 4). Main imaging stages including image acquisition and processing have great impacts on radiomic feature values. Previous studies have shown radiomic features vary against image acquisition parameters, reconstruction, slice thickness, matrix size and type of scanner (5, 6). Moreover, robust features against challenging parameters have been identified.

By introducing imaging biobanks for image biomarker sharing, radiomic science has entered into the new era. “They are defined as organized databases of medical images, and associated imaging biomarkers shared among multiple researchers, linked to other biorepositories (7)”. In regard to imaging biobanks, “it is possible to implement platforms that allow for the combination of imaging biomarker analysis with big data capabilities for the assessment of quantitative exploitation of knowledge, not limited to imaging and pooled with other environmental, clinical, and omics’ information of the patients. These kinds of solutions can be used for management of diseases, such as detection and treatment response evaluation” (8).

In imaging departments, medical imaging technologists (MITs) including radiology, MRI, CT, SPECT and PET technologists have central role in all imaging processes. Although the Medical Physicists play a critical role, technologists are in the front line of image science. They do determine how image can be acquired, how image can be processed and displayed.

In regard to the quantitative imaging, radiomics, and imaging biobanks, and due to lack of knowledge on their direct and great impacts on final radiomics results, MITs may be confused and do their acts unartfully. Because MITs have been trained for classic routine qualitative imaging, they may suffer from understanding new quantitative imaging approaches e.g. radiomics. In this condition, to obtain best radiomics outcome, MITs have to be trained in the radiomics specific concepts, policies, procedures, technologies, and know-how of an MIT to help perform their duties efficiently.

**Fig. 1:**
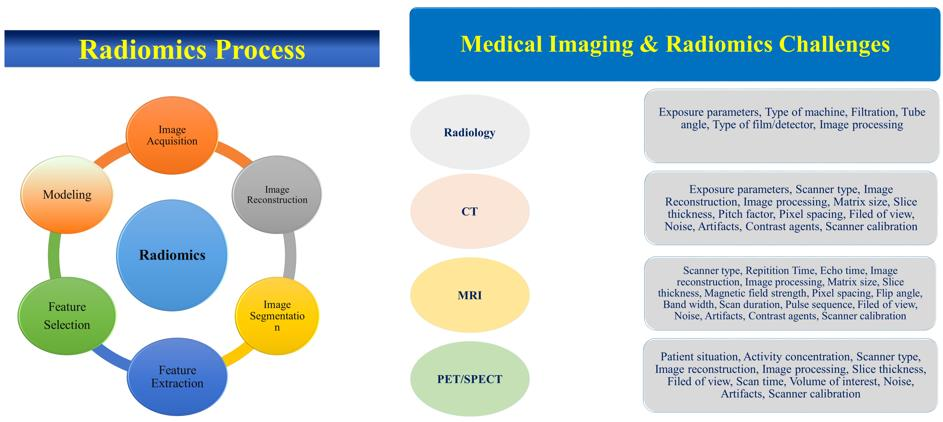
The main challenging parameters which have great impacts on radiomics outcome

In Fig. 1, we summarized the main challenging parameters which have great impacts on radiomics outcome. An MIT encounters different challenging parameters which directly or indirectly changes the feature values and therefore the final radiomics results. Based on the imaging modality, the challenging parameters could be changed. MITs have to be trained to do the best and optimized imaging protocols. On the other hand, because MITs are more familiar with imaging machines, protocols and daily routine experiments they may have good offers for optimized imaging protocols and therefore best radiomics results. Moreover, for data sharing as per suggested by imaging biobanks, there must be a consensus among MITs to obtain best radiomic results. Imaging scientists may contribute for MITs training.

Finally, although this opinion is free of experimental data, MITs have a critical role in radiomics results and their high knowledge and attitude may contribute to more optimized and effective radiomics outcomes. Feasible knowledge on radiomics aim, radiomic features, feature robustness, radiomic process and challenges, imaging protocols and processing will resulted in MITs best works on radiomics.
